# A Case of Tuberculous Meningitis with Tuberculoma in Nonimmunocompromised Immigrant

**DOI:** 10.1155/2016/9016142

**Published:** 2016-06-20

**Authors:** Parth Rali, Hammad Arshad, Eric Bihler

**Affiliations:** Division of Pulmonary and Critical Care, Allegheny General Hospital, 320 E. North Avenue, Pittsburgh, PA 15212, USA

## Abstract

We present a case of tuberculous (TB) meningitis in nonimmunocompromised immigrant worker who initially presented with headache and later with generalized tonic clonic seizures and disseminated tuberculosis.

## 1. Introduction

Tuberculosis is one of the most common infections in developing countries. Tuberculosis usually presents as primary pulmonary disease. It can present as disseminated form with involvement of central nervous system being very often. Tuberculoma is a unique finding seen in patients with TB meningitis. Tuberculomas can be present at diagnosis or develop during treatment.

## 2. Case Summary

**Figure 1:**
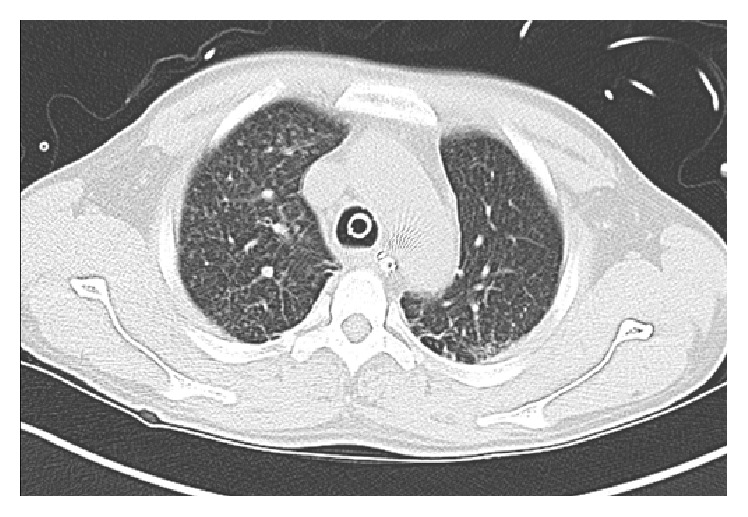
Chest CT scan showing numerous randomly distributed nodules bilaterally in upper lobe consistent with miliary pattern of distribution.

**Figure 2:**
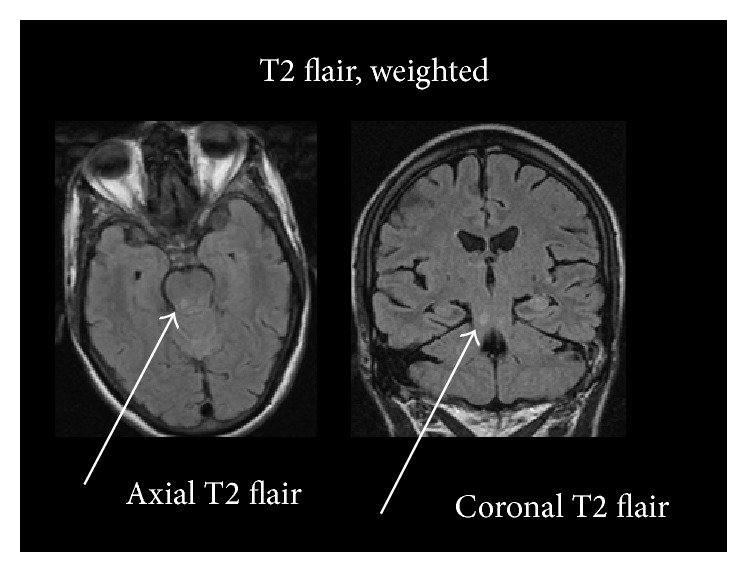
Images show T2 hyperintense focus in the posterior aspect of the midbrain-pons junction.

**Figure 3:**
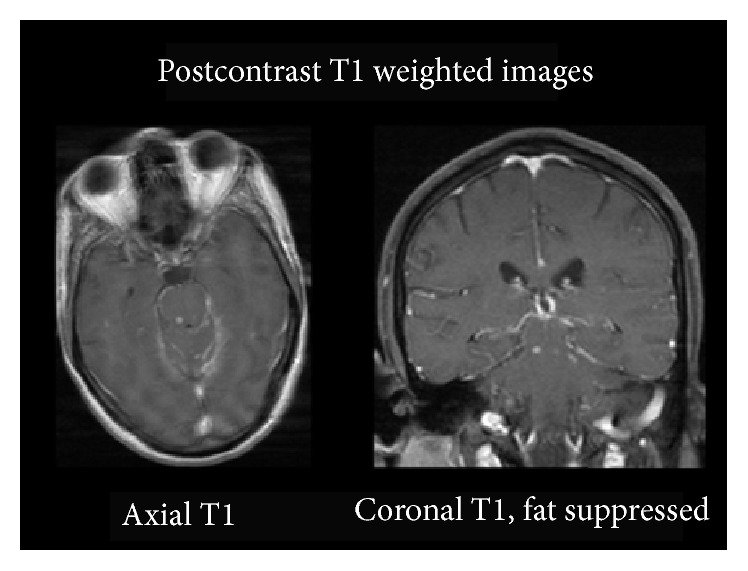
Postcontrast T1 weighted images. Enhancement of the same T2 hyperintense lesion can be seen. The axial image shows enhancement of the basilar meninges which is also commonly seen in TB.

**Figure 4:**
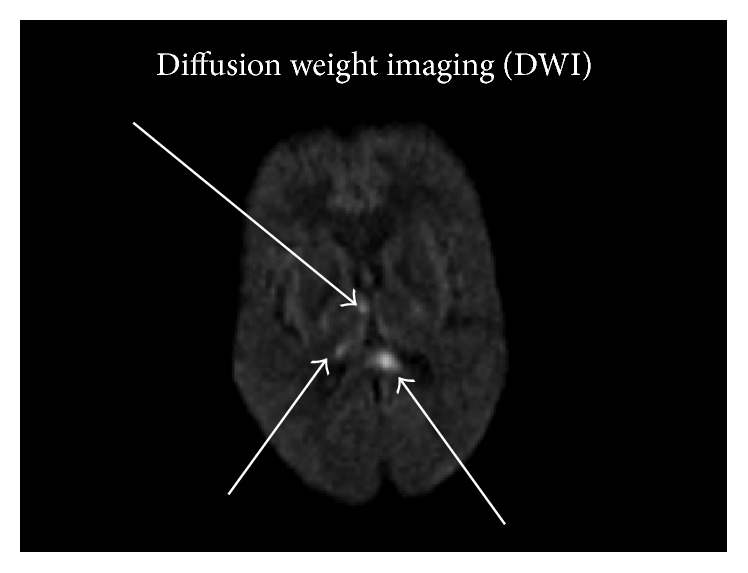
DWI shows bright areas (restricted diffusion) in the splenium of the corpus callosum and right thalamus compatible with infarcts. These areas were low on the apparent diffusion coefficient maps which confirms that these are infarcts (not shown). Infarcts are a known complication of neuro-TB.

The case was that of a 45-year-old immigrant from Nepal who presented to emergency department with headache, agitation, altered mental status, and productive cough of two weeks' duration. Patient had moved to United States 2 and a half years ago. He was vaccinated with BCG as a child. He was screened negative for latent TB at the time of his immigration. He did not have any recent travel or TB exposure. He worked in a restaurant. Patient did not have any medical problems. There was no family history of TB. In emergency room, he underwent CT of the head. CT of the head was negative for acute disease and patient subsequently underwent lumbar puncture (LP). Cerebrospinal fluid (CSF) showed lymphocyte predominance and a positive DNA probe for MTB on CSF which was susceptible to standard first-line treatment. His initial sputum cultures were negative for AFB, but BAL obtained via bronchoscopy grew* Mycobacterium tuberculosis* (MTB). He was discharged on RIPE (rifampin, isoniazid, pyrazinamide, and ethambutol) treatment and instructed to follow up at the health department. A few days later, the patient again presented to the hospital after having a tonic clonic seizure and required intubation for hypoxemic respiratory failure (Figure 1). On physical examination, the patient was found to have unequal pupils which prompted repeat neuroimaging. On MRI, tuberculomas were observed diffusely including the posterior midbrain affecting the cranial III nucleus accounting for the anisocoria (Figures 2, 3, and 4). Due to visual disturbances, patient was also evaluated by ophthalmology and was found to have severe optic neuritis. Levaquin was substituted for ethambutol and prednisone was added to isoniazid, rifampin, and pyrazinamide. Over the next 1-2 weeks, patient significantly improved and was subsequently discharged for total duration of 18 months of antituberculous treatment. He received prednisone for first 4 weeks' duration. All subsequent sputum cultures remain negative for MTB on follow-up. His vision returned to baseline in the next 6 months. Patient had no neurological deficits after completion of his treatment. Three of his family members were diagnosed with latent tuberculosis which were treated with 9 months of INH therapy.

## 3. Case Discussion

Tuberculous meningitis (TBM) accounts for 2–5% of all active cases of MTB [1]. Coexisting pulmonary disease is seen in 25–83% of TBM [2–4]. Primary CNS disease is predominately found in children. TBM is categorized based on location of disease on imaging into leptomeningeal, parenchymal, or other involvement. Leptomeningeal involvement presents as meningitis, cranial nerve (CN) palsies (most commonly CN 2, 3, 4, and 7), and communicating hydrocephalus. Leptomeningeal involvement results from either hematogenous spread or rupture of subpial or subependymal foci (known as Rich foci) into the subarachnoid space [5]. Parenchymal disease is categorized into tuberculomas with or without meningitis and rarely brain abscess.

Tuberculomas are organized clusters of inflammatory cells meant to limit the spread of* Mycobacterium* bacilli. They may be present on initial imaging or develop during treatment. Hosoglu et al. found that tuberculomas are present on 8% of CT of the head performed on patients with TBM [6]. MRI has been found to be superior to CT of the head for visualization of tuberculomas. Multiple lesions are found, typically supratentorial, more common than solitary tuberculomas [7]. Based on imaging tuberculomas can be categorized into noncaseating, caseating with solid center, or caseating with a liquid center. High CSF protein >3 g/L has been associated with increased incidence. Clinical deterioration secondary to tuberculoma expansion during treatment has been described and is termed a paradoxical reaction [8].

Diagnosis of TBM can be challenging and large volume LP is needed. CSF analysis typically demonstrates lymphocyte predominate pleocytosis (100–500 cells/microliter), elevated protein (100–500 mg/dL), and low glucose (usually less than 45 mg/dL). Sensitivity for single CSF analysis for AFB smear is low around 20–40%. Nucleic acid amplification has a sensitivity of 56% and specificity of 98%. Multiple lumber punctures are often needed for diagnosis.

Treatment TBM is similar to that of other forms of tuberculosis. There is an initial phase consisting of four drug regimens; however the continuation phase is often extended to complete 9–12 months of therapy. Even though quinolones have excellent CNS penetration, they are known to aggravate seizures and should be avoided in patients with documented seizures. Streptomycin is a better choice in those cases. A Cochrane meta-analysis of seven RCT compiling 1,000 pts concluded that corticosteroids improve outcomes [9]. 6–8 weeks' course of steroids should be added in the beginning along with antituberculous treatment to avoid complications like cranial nerve palsies, stroke, and seizures. In patients with significant paradoxical reactions, corticosteroids are sometimes maintained throughout treatment. Obstructive hydrocephalus requires surgical intervention, in the form of VP shunt or third ventriculostomy. The presence of tuberculomas on presentation, formation during treatment, or paradoxical reactions do not seem to affect the prognosis of TBM [7].
